# Spectroscopic Detection of Caries Lesions

**DOI:** 10.1155/2013/161090

**Published:** 2013-01-08

**Authors:** Mika Ruohonen, Katri Palo, Jarmo Alander

**Affiliations:** ^1^Faculty of Technology, University of Vaasa, P.O. Box 700, 65101 Vaasa, Finland; ^2^Dental Services of the City of Vaasa, Social and Health Administration, P.O. Box 241, 65101 Vaasa, Finland

## Abstract

*Background*. A caries lesion causes changes in the optical properties of the affected tissue. Currently a caries lesion can be detected only at a relatively late stage of development. Caries diagnosis also suffers from high interobserver variance. *Methods*. This is a pilot study to test the suitability of an optical diffuse reflectance spectroscopy for caries diagnosis. Reflectance visible/near-infrared spectroscopy (VIS/NIRS) was used to measure caries lesions and healthy enamel on extracted human teeth. The results were analysed with a computational algorithm in order to find a rule-based classification method to detect caries lesions. *Results*. The classification indicated that the measured points of enamel could be assigned to one of three classes: healthy enamel, a caries lesion, and stained healthy enamel. The features that enabled this were consistent with theory. *Conclusions*. It seems that spectroscopic measurements can help to reduce false positives at *in vitro* setting. However, further research is required to evaluate the strength of the evidence for the method's performance.

## 1. Introduction

 Minimally invasive dentistry is an approach that seeks to maintain the patient's oral health with preventive measures and to treat possible disturbances of health as early as possible and with as little intervention as possible [1]. This requires that caries is detected at an early stage of development and that its status can be monitored frequently [2]. However, the current methods for diagnosing caries are able to detect caries only at a relatively advanced stage. Accordingly, methods for early detection of caries have been researched for the past twenty years. Many of these methods still require extensive research before they can be used in clinical practice. Optical caries diagnosis methods are based on the fact that caries cause changes in the tooth's optical properties at an early stage of development [3].

This was a pilot study to investigate whether diffuse reflectance visible/near-infrared spectroscopy (VIS/NIR-S) can be used to detect dental caries lesions. Reflectance spectroscopy measures the intensity of light at several different wavelengths, that is, its spectra, after the light has reflected from the studied object. Diffuse reflectance refers to light that has been reflected from the inside of the object, rather than from its surface. In this study the intensity was measured at wavelengths in the visible range and at wavelengths in the near-infrared range, covering wavelengths in the range 420–1000 nanometers. Within this range, the intensity was measured at 2305 different wavelengths, so that the difference between consecutive wavelengths was approximately 0.25 nm. This study was limited to studying natural caries lesions that could be diagnosed with fiber-optic illumination, on smooth surfaces of extracted tooth.

A theory of caries diagnosis using near-infrared spectroscopy emerges from the previous studies of detecting caries lesions with near-infrared light [2–7]. According to this theory, the development of a caries lesion increases the porosity of the affected tissue, which in turn leads to an increased scattering of light in the lesion. Wavelengths in the near-infrared range are considered better than the wavelengths in the visible range, because the former can penetrate deeper into the tissue and are less affected by stains on the tooth. The purpose of this study was to provide additional evidence in support of this theory. More work on this topic can be found in [8–15].

## 2. Methods

### 2.1. Samples

The dental services of the City of Vaasa provided extracted human teeth for the study. The teeth were stored immersed in denatured alcohol in order to disinfect them and to keep them hydrated. Before inspection and measurements, the teeth were gently dried with a cue tip. The teeth were inspected by the first author with fiber-optic illumination, after the technique was introduced to him by the second author, in order to detect healthy areas of enamel and areas of enamel that contained caries lesions.

In total 21 teeth were used in the study. A total of 109 points of enamel were measured on the teeth, consisting of 69 points which were thought to represent healthy enamel and 40 points which were thought to represent caries lesions. Each measurement point produced a spectra, a sample for the rest of the analysis. In pattern recognition terminology the diagnosis of a given sample, as either healthy or carious, is called the label of the sample. The analysis of the samples tries to create a method which estimates the diagnosis, the label, of the sample based only on the measurements. The resulting estimates are called predictions.

### 2.2. Measurements

**Figure 1:**
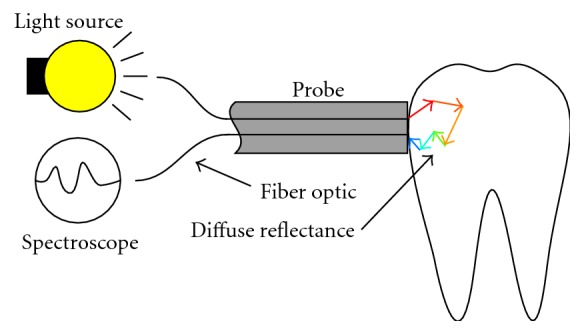
An illustration of the measurement setup.

The measurement setup is presented in Figure 1. An optical fiber, placed in contact with the sample, conveys light from a light source to the sample. The light enters the sample and scatters to all directions inside of it. Another optical fiber is placed in contact with the sample at a small distance from the first fiber. Some fraction of the light which scatters inside the sample will eventually exit the sample so that it enters the second optical fiber. It then gets conveyed to a spectrometer, which measures the spectra of the reflected light. Properties of the sample material affect the measured spectra. Photonics describes the key properties with the absorption coefficient and the scattering coefficient of the material. The measured spectra is analyzed in order to deduce information about the sample material. 

The measurements were made with a spectrometer HR4000 (Ocean Optics Inc., Dunedin, FL, USA) and with a general purpose transmission dip probe model T300-RT-VIS/NIR (Ocean Optics Inc., Dunedin, FL, USA). The probe contains two optical fibers, both with a diameter of 300 *μ*m, housed in a stainless steel assembly with a diameter of 3.175 mm. The assembly is surrounded by a ferrule with a diameter of 6.35 mm. One of the fibers is connected to a light source and brings light to the sample. The light source used in this study was a tungsten halogen lamp HL-2000 (Ocean Optics Inc., Dunedin, FL, USA). The other fiber is connected to the spectrometer. It collects and transmits the diffusely reflected light. Construction of a custom probe for this study was deemed unfeasible. Thus, the study had to be carried out with a probe that was readily available in our laboratory. The selected probe is designed for measuring the transmission spectra of liquid samples. However, it was considered to be suitable for this study when the ferrule enclosing the inner assembly was removed, exposing the stainless steel assembly that houses the fiber optics.

The period of time for which the spectrometer collects light when it is making one measurement is called the integration time. In this study integration time was set to 20 milliseconds. A longer integration time produces better measurement results than a short one, because the intensity of the collected light increases at all wavelengths, yielding a better signal-to-noise ratio (SNR). Therefore, the integration time is typically set as long as possible. However, if the intensity of the collected light at a given wavelength exceeds the measurement range of the spectrometer, the spectrometer saturates. In that case the intensity cannot be measured, and we know only that it exceeded the maximum measurable value.

In order to make the measurement results comparable to results that would have been obtained with the same spectroscope using another light source or another integration time, the spectroscope has to be calibrated for these factors. This is done by measuring the smallest and the greatest intensity value that a measurement can produce with the given integration time for each wavelength and by scaling all other measurements to that range. This gives values between zero and one for all wavelengths. These scaled results are called normalized intensities. The lowest possible intensity values are obtained by measuring the so-called dark current, which is caused by thermal noise. Measuring a white reference sample produces the greatest possible intensity values. In this study, the integration time was set so that the white reference sample (a white reference tile WS-2, Avantes Inc., Eerbeek, The Netherlands) did not saturate at any wavelength. A spectrometer must also be calibrated for its detector, so that its measurement results are comparable to those obtained by other spectroscopes. This is done by measuring the spectra of a sample whose spectra is known. In this study the used spectroscope was calibrated for its detector by the manufacturer as part of its construction.

A spectroscopic measurement result contains many small random errors, which are collectively called (thermal) noise. These errors are caused by heat, or thermal energy, in the spectroscope. They follow a normal distribution with a given mean value. The dark current presents the mean value of the noise for each wavelength. When the dark current is subtracted from the spectra, the mean value of the effect caused by the noise is shifted to zero, and thus the effect of noise is observed as errors which have a normal distribution with a zero mean. In order to minimize the effect of noise in the samples, each point was measured one hundred times consecutively, and the resulting spectra were averaged. This meant that the probe needed to stay as motionless as possible for two seconds. However, a far shorter time period would have probably been sufficient.

### 2.3. Analysis

As a further measure against noise, the samples were smoothed by using the Savitzky-Golay method with a window length of 61 and sixth degree polynomials. This method selects the coefficients of a sixth degree polynomial so that the polynomial is the best possible approximation for the measurement result, that is, the spectra, for the 30 wavelengths before a given wavelength and for the 30 wavelengths after it. The value of the polynomial at the given wavelength replaces the measured intensity at that wavelength. This removes, or smoothens, fast and small changes in the spectra, which are mainly caused by noise.

A simple computational algorithm, based on exhaustive search, was then used to find a set of rules that could be used for detecting caries lesions. At this point, the goal was to classify the samples into two classes: points on healthy enamel (healthy samples) and points on caries lesions (carious samples). For this, a set of rules was searched for, so that every rule had the following format: if the sample's normalized intensity at a given wavelength *λ* is greater than (or smaller than) a given threshold *I* ^⋆^, the sample is classified as carious; otherwise, the sample is classified as healthy. Thus, each rule had three parameters: the wavelength *λ*, the intensity threshold *I* ^⋆^, and whether or not the threshold is an upper or lower limit for the intensity. If, and only if, one or more of the rules classified the sample as carious, the sample was classified as carious. If none of the rules considered the sample as carious, it was classified as healthy. A pseudocode for this step is given in Pseudocode 1. It was hoped that the algorithm would select a set of rules which resembles the results found in earlier studies on this subject.

A number of wavelengths were selected from the range of available wavelengths (*≈*420–1000 nm) as options for parameter *λ* in the search, so that the intervals between the wavelengths were equal and the first and the last wavelength were always selected as options. A pseudo-code for this is given in Pseudocode 2. The search was done with different numbers of wavelengths. For each of the selected wavelengths, the algorithm sorted the samples' intensities at that wavelength and considered the midpoint between each two consecutive intensities as a possible threshold *I* ^⋆^ in a rule. A pseudo-code for this is given in Pseudocode 3.

The algorithm calculated the classification accuracy on the training set for each of the pairs *λ* and *I* ^⋆^ described above, using the threshold *I* ^⋆^ first as an upper limit for classifying the sample as carious and then using it as a lower limit, and chose the values of *λ* and *I* ^⋆^ and the type of threshold, which gave the best accuracy (see pseudo-code at Pseudocode 4). After a rule had been selected this way, the algorithm selected another rule with the same method, so that the new rule gave the best possible accuracy when used together with the previously selected rule(s). This was continued until the maximum allowed number of rules, here five rules, was reached, or until the classifier was unable to find a new rule which would improve the classification accuracy. A pseudo-code for this logic is given in Pseudocode 5.

This algorithm, like every machine learning method, requires a set of samples which is used for searching for the rules and a separate set of samples which is used for evaluating the accuracy that is achieved with the resulting rules. The former set of samples is called the training set and the latter set is called the validation set. The number of samples available for this study was rather limited. This may cause problems for the machine learning method when the samples are divided into a training set and a validation set, because some types of samples may become overrepresented in the training set, misleading the learning method as it tries to recognize what discerns the two classes from each other.

In this study, this risk was alleviated by using a 4-fold cross-validation. In this method, the samples are divided into four groups, and one of them is used as the validation set while the other three groups form the training set. Each group in turn is used as the validation set, and the results from these four “folders” are averaged. This way each sample is a part of the training set in three folders and a part of the validation set in one folder. It is unlikely that the same types of samples would be overrepresented in all four training sets, unless the entire set of available samples has this problem. A single training set which has this problem would stand out from the others, and the skewed learning results from it would be corrected by the results from the other training sets. While the small set of samples may still give a skewed representation of the kinds of samples which are being studied, the cross-validation seeks to minimize this problem.

In this study the averaging was done so, that a median rule set was constructed from the rules which the algorithm selected for the folders, and all of the samples were then classified with the median rule set. Median of the numbers of rules in the folders determined the number of rules in the median rule set. Some manual deliberation was used when constructing the rules of the set. For each rule in the final set, a temporary rule set was composed by selecting one rule from each folder's rule set, so that the rules in the temporary set resembled each other, if that was possible given the available rules. The median of the wavelengths used in the rules in the temporary rule set determined the wavelength for the rule in the median rule set. The intensity threshold and the type of threshold were selected similarly for the rule in the median rule set. A pseudo-code for this is given in Pseudocode 6. 

Each sample was diagnosed as either healthy or carious by the first author, and the selected rules estimated each sample to be either healthy or carious. Based on these two properties, the samples can be divided into four classes. Samples which were diagnosed as healthy and which were estimated to be healthy by the rules are called true negatives (TNs). Similarly, carious samples which were correctly estimated are called true positives (TPs). A healthy sample which was estimated to be carious is called false positive (FP) and a carious sample which was estimated to be healthy is called a false negative (FN). The sizes of these classes comprise a confusion matrix, or a contingency table. These four values can be used to calculate the following five values which describe the accuracy of the selected rules.

- Positive predictive value (PPV) is the probability that the classifier, that is, the set of rules, is correct when it estimates a sample to be carious.
- Negative predictive value (NPV) is the probability that a healthy estimate is correct.
- Sensitivity is the fraction of all carious samples that were classified as carious.
- Specificity is the fraction of healthy samples that were classified as healthy.
- Accuracy is the fraction of the samples which were correctly estimated, that is, where the rules gave the correct answer.

### 2.4. Two Hypotheses of Misdiagnosis

After the classification rules had been selected and the samples had been classified according to them, there were fifteen samples which the author had diagnosed as carious but which were classified as healthy (false negatives). The spectra of these samples were virtually indistinguishable from the spectra of the healthy samples (see Figure 3(a)), at least for the analysis methods used in this study. Thus, a hypothesis was made that these samples, the false negative cases, had been misdiagnosed by the author and subsequently mislabeled.

The rules that were selected by the algorithm suggested that a short wavelength, namely, 420 nm, was relatively useful in the diagnosis of caries. This was inconsistent with the theory on the optical diagnosis of caries. Therefore, another hypothesis was made, according to which a number of samples had been diagnosed by the author as carious while in fact the measured points were only stained and were thus false positive cases of the diagnosis, even if they had been classified correctly by the classifier. A pair of rules was manually selected in order to detect such stained samples. These rules were *I*(*λ* ≈ 420) ≤ 0.206∧*I*(*λ* ≈ 815) ≤ 0.313. Notation *I*(*λ*) refers to the normalized intensity of the spectra at wavelength *λ*. In other words, the sample was thought to represent a stain if it had a small scattering coefficient at both a long wavelength (815 nm, which is in the near-infrared range) and a short wavelength (420 nm). Application of these rules identified eight samples as being misdiagnosed due to a stain.

## 3. Results

**Figure 2:**
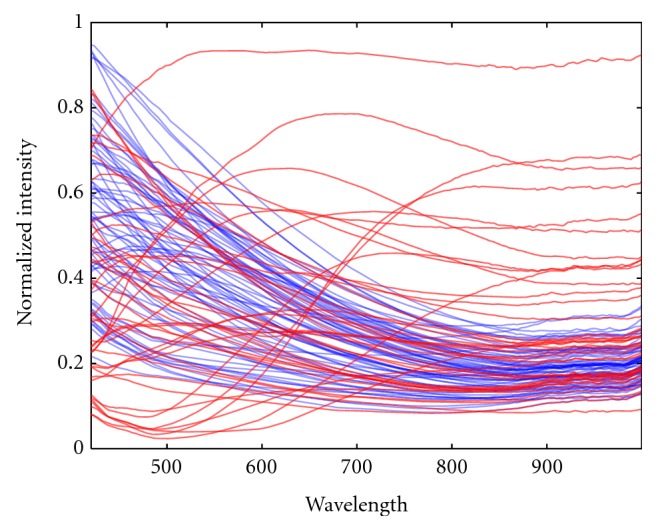
The samples, that is, the spectra of the measured points. The blue curves depict samples which were diagnosed as healthy and the red curves depict samples which were diagnosed as carious.

The samples, or the spectra of the measured points, are presented in Figure 2. The number of wavelengths which were selected as options for the rule's parameter *λ*, that is, parameter W avelen gth OptionCount, had only a small effect on the accuracy of the resulting median rule set. When only the shortest wavelength (*≈*420 nm) and the longest wavelength (*≈*1000 nm) were available as options, the median rule set had an accuracy of 82%. With three wavelengths to choose from, the accuracy was 83%. When the number of options was between four and six, the accuracy was 85%. With greater numbers of wavelengths available, the accuracy was 84%.

The selected rules were very similar in all folders. This suggested that the rules depicted a phenomenon which was consistently present in all four folders. When the number of options for the rules' wavelengths was 15, the median rule set indicated that a sample is carious if, and only if, *I*(*λ* ≈ 420) ≤ 0.2642∨*I*(*λ* ≈ 750) ≥ 0.3502. A confusion matrix of the classification accuracy that is achieved with these rules is presented in Table 1, showing that these rules reached an accuracy of 84%. 

**Figure 3:**
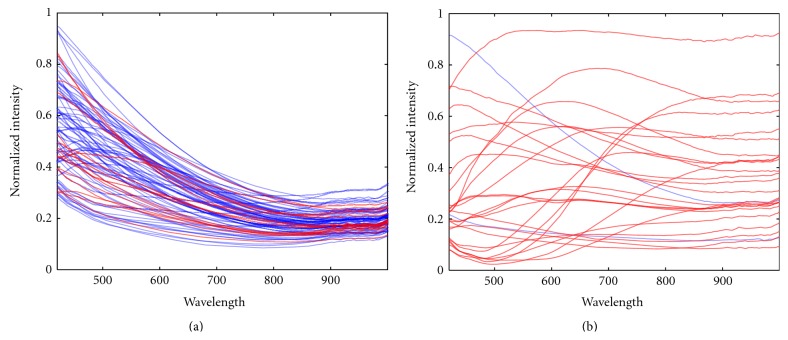
Samples which were classified (a) as healthy and (b) as carious by the median rule set. Blue curves represent healthy samples and red curves represent carious samples. The samples which were diagnosed as carious but classified as healthy (false negatives) are emphasized.

As can be seen in Figure 3(a) and in Table 1, there were fifteen carious samples which were classified as healthy (false negatives), and whose spectra was virtually indistinguishable from the spectra of the healthy samples. As explained in Section 2.4, this leads to a hypothesis that these samples had been misdiagnosed and subsequently mislabeled, and that they therefore represented healthy samples and were in fact classified correctly. 

According to the theory on optical caries diagnosis, an elevated intensity in the near-infrared range is the best indication of a dental caries lesion. However, the rules selected by the search algorithm indicated that a short wavelength, namely, 420 nm, was relatively useful in the diagnosis of caries. As explained in Section 2.4, another hypothesis was thus made, according to which a number of samples had been diagnosed as carious while in fact they were only stained. A pair of rules was manually selected in order to detect such stained samples.

Application of these rules identified eight samples as being misdiagnosed due to a stain. All samples that were identified as stained had been diagnosed and classified as carious, and thus appeared to be true positive cases. These suspected misdiagnoses had not lowered the apparent accuracy of the classification, but they may have caused the rule set to erroneously consider stains as caries lesions.

When the search algorithm was run again, giving 15 options for the parameter *λ*, after first relabeling the fifteen false negative cases as healthy samples (first hypothesis) and then relabeling the eight suspected stains as healthy samples (second hypothesis), the algorithm selected only one rule in every cross-validation folder. All rules set an upper limit for the normalized intensity at a wavelength in the near-infrared range. If the intensity was greater than this, the sample was classified as carious. The median of those rules was *I*(*λ* ≈ 791) ≥ 0.3255, which is consistent with the theory. The confusion matrix of this rule is presented in Table 2. This rule produced an accuracy of 97%.

## 4. Discussion

This study suffers from a small number of samples. Although the study used 109 measurements, they were taken from only 21 individual teeth. This fact is significant, because it is probable that samples taken from a single tooth resemble each other more than samples taken from different teeth or from different patients. Furthermore, the 109 measurements contained only 40 measurements from a caries lesion. Fifteen of those measurements were considered to be misdiagnosed by the first hypothesis, and further eight measurements were considered to be misdiagnosed by the second hypothesis. Therefore, further study is needed to increase the reliability of the accuracy estimate of this method.

The measurement results together with the theory on the topic suggest that many of the measurements which were supposedly made from a caries lesion are in fact made from healthy enamel, which was in some cases stained. When we make these suggested corrections to the labeling of the samples, the samples seem to fit well to the theory and the samples can easily be accurately classified. These kinds of diagnostic mistakes, or false positive diagnoses, are a credible explanation, because the diagnoses were made by a novice on the subject. However, such corrections also pose a risk that the measurement results are relabeled to make them fit the theory, which would inflate the accuracy of the method. Further study of the method might dispel such possibilities.

The composition of the dental tissues varies from tooth to tooth and between different sites of a given tooth [16]. As can be seen in Figure 2, the spectra of the different healthy samples vary quite a bit, especially at the visible wavelengths. This suggests that the threshold intensity or intensities for diagnosing a suspected lesion as carious might also vary similarly. In order to compensate for the inter-tooth and intra-tooth variance, we might consider measuring the average spectra for a given tooth by measuring several points on the tooth surface, that is, by scanning the surface and by evaluating how much the spectra of the suspected lesion differ from the tooth's average.

Unfortunately, this approach could potentially make this method less effective for its original purpose. The method is being developed for the detection of caries lesions at an early stage of development. Thus, the dentist does not necessarily notice all of the lesions which are detected by the device. If the inspected tooth surface contains several developing caries lesions, the average spectra of the surface could be something in between the healthy enamel and the carious enamel, making the lesions appear too similar to the average surface to be diagnosed as carious. A set of fixed thresholds would avoid this problem. The scanning method would also make it rather awkward to inspect several teeth per patient.

Quantitative Light-induced Fluorescence (QLF) and Laser-induced Fluorescence (LF) are two optical methods for the detection of caries lesions. They are based on fluorescence, or the phenomenon that when the tooth sample is illuminated with a light source, some of the light is absorbed in the sample, after which the sample emits light at a longer wavelength. For both methods the emitted wavelength falls within the range of measured wavelengths [3]. In this study the sample was considered carious if the measured intensity was greater than a fixed threshold, *I*(*λ* ≈ 791) ≥ 0.3255. The proposed explanation is that the increased scattering due to caries causes more light to be reflected to the measuring fiber optic. QLF expects to find a reduced intensity for carious samples at wavelengths *λ* > 520 nm because increased scattering due to caries interferes with the detection of the fluorescence, and LF expects to find increased intensity at the near-infrared range caused by fluorescence from organic molecules in the sample [3].

Since the samples in this study were stored in denatured alcohol, they were probably relatively free of organic molecules. Further study is required to determine whether the fluorescence from organic molecules, that is, the phenomenon measured by LF, interferes with the detection method outlined in this study, especially for *in vivo* measurements. If it does interfere, it probably makes the method more eager to label a sample as carious, thus increasing its sensitivity and reducing its specificity. This effect may be modified, at least in part, by selecting a new set of rules based on results from *in vivo* measurements. Incidentally, low specificity has been cited as a major weakness of the LF method [3]. In contrast, authors of this study felt that the method outlined in this paper helped them to increase specificity.

An ability to measure the amount of dental tissue lost to caries could be pursued by inducing caries *in vitro* to a tooth sample (see [6, 17, 18]) so that the amount of mineral dissolved from the tooth could be measured without destroying the sample, and by measuring the spectra of the sample at varying degrees of mineral loss. One possible method for this would be to cycle the tooth sample in de- and remineralization solutions and to measure the amount of mineral dissolved to the solutions with a mass spectrometer. This would have to be repeated with a sufficient number of samples. Finding a method to calculate the amount of the mineral loss from the spectra would be a regression problem. 

## 5. Conclusions

It seems that spectroscopic measurements can help to reduce false positives at *in vitro* setting, including those caused by stains. This method may also give objective evidence of the presence of a caries lesion. However, the work reported in this paper was a pilot study, and further research is required to evaluate the strength of the evidence for the method's performance at *in vitro* setting and to extend the measurements to *in vivo* setting.

## Figures and Tables

**Pseudocode 1:**
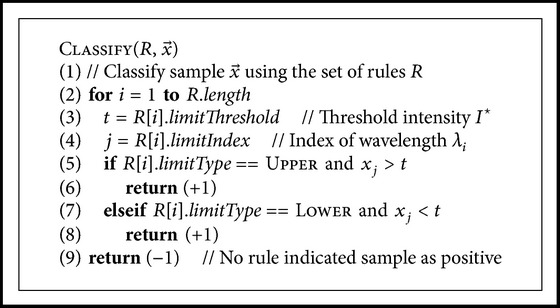
Pseudocode for classifying a sample. Samples in the positive class are carious, and samples in the negative class are healthy. A sample $\vec{x}$ is a vector, where each component *x*_*i*_  equals the normalized intensity at a given wavelength *λ*_*i*_.

**Pseudocode 2:**
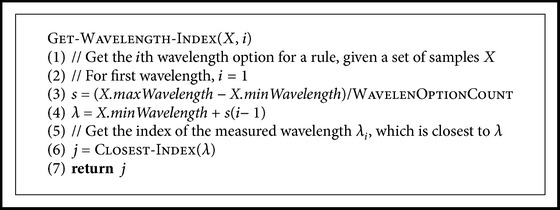
Pseudocode for computing the *i*th wavelength option for a rule.

**Pseudocode 3:**
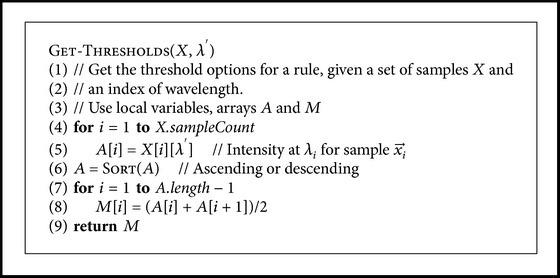
Pseudocode for computing the threshold options for a rule at a given measured wavelength *λ*_*i*_. The wavelength is defined by its index, *λ*′ = i.

**Pseudocode 4:**
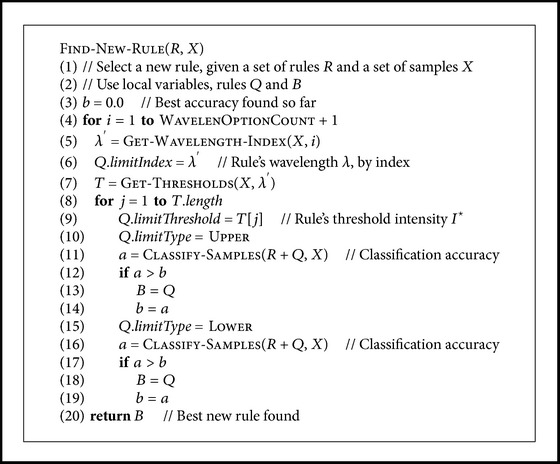
Pseudocode for selecting a new rule.

**Pseudocode 5:**
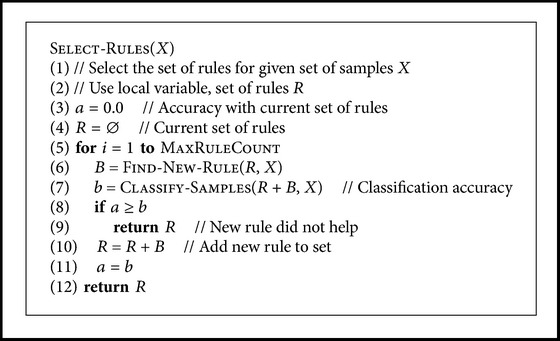
Pseudocode for selecting the set of rules. Here *X* is the set of training samples and Ma xRuleCount = 5.

**Pseudocode 6:**
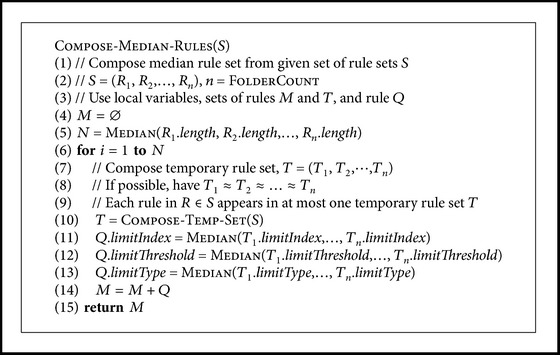
Pseudocode for computing the median rule set. In this study Fo lderCount = 4.

**Table 1:** The confusion matrix, or the contingency table, of the median rule set.

	Carious	Healthy	
Estimated carious	25 (TP)	2 (FP)	93% (PPV)
Estimated healthy	15 (FN)	67 (TN)	82% (NPV)

	63% (Sens.)	97% (Spec.)	84% (Acc.)

**Table 2:** The confusion matrix, or the contingency table, for the median rule (set) which was selected after relabeling the samples according to the two hypotheses of misdiagnosis.

	Carious	Healthy	
Estimated carious	14 (TP)	0 (FP)	100% (PPV)
Estimated healthy	3 (FN)	92 (TN)	97% (NPV)

	82% (Sens.)	100% (Spec.)	97% (Acc.)
